# Evaluation of the accuracy of implant placement by using implant positional guide versus freehand: a prospective clinical study

**DOI:** 10.1186/s40729-023-00512-z

**Published:** 2023-12-01

**Authors:** Lixuan Huang, Linfeng Liu, Shengtao Yang, Prapti Khadka, Shiwen Zhang

**Affiliations:** 1 State Key Laboratory of Oral Diseases and National Center for Stomatology and National Clinical Research Center for Oral Diseases, Chengdu, People’s Republic of China; 2 Department of Oral Implantology, West China Hospital of Stomatology, Sichuan University, Chengdu, People’s Republic of China

**Keywords:** Dental implant, Surgical guide, Implant positional guide, Implant accuracy

## Abstract

**Purpose:**

The aim of this study is to examine and compare the accuracy of implant placement using implant positional guide and freehand.

**Methods:**

48 implants were placed in patients with single tooth loss with implant positional guide and freehand, respectively. The accuracy of implant placement was assessed by comparing the actual and planned position, including four parameters: coronal deviation, apical deviation, angular deviation, and vertical deviation.

**Results:**

Comparing all the variables, it has been found that the implant positional guide is more accurate than the freehand. All parameters describing in the deviation were significantly lower in the implant positional guide group than the freehand.

**Conclusions:**

The implant positional guide can act as a practicable tool for dental implant surgery. It is a promising technology that guarantees low cost and high precision in implant surgery. However, based on the restricted evidence from clinical studies, longer follow-up periods, larger population studies, and standardized experimental studies are required.

*Trial registration* CHICTR, ChiCTR2300071024. Registered 28 April 2023—CHICTR, ChiCTR2300071024. Registered 28 April 2023—Retrospectively registered, https://www.chictr.org.cn/showproj.html?proj=195424.

## Introduction

Precise implant placement is very dependent on the experience of the surgeon [1–3] and needs to be verified repeatedly during the surgical procedure. Failures do occur. Sometimes it is necessary to remove the implant, sometimes it may jeopardize the possibility of achieving a satisfactory restoration, both functionally and esthetically [4–7]. Moreover, failures can entail additional expenses and more complex procedures for the patients.

Digital technology is increasingly used in the multidisciplinary dental treatment process, realizing a precise, efficient, minimally invasive, repeatable process and predictable results, which is undoubtedly the trend of future dental treatments [8, 9]. At the same time, the concept of restoration-oriented implantation is gradually being accepted by more and more clinicians. To obtain successful restorative results, precise preoperative planning is essential to avoid anatomical structures and to place the implants in a way that produces an esthetically pleasing restoration [10–13]. Personalized preoperative diagnostic design and oral implant surgical guides have become essential for transferring the preoperatively designed implant placement to the implant surgery [14].

Implant surgical guides can be classified into fully guided and partially guided surgical guides. Fully guided surgical guides have an entirely restrictive procedure, where the movement of the drill is restricted during the entire drilling and implant placing process. The partially guided guide is removed in advance, the final drilling and implant placement is performed freely [15]. Although the implant surgical guides allow for precise implant placement, the procedure is complex and requires a guided surgery cassette and specialized training for the clinician [16]. Most critically, it greatly increases the cost to the patient, the cost to the hospital for cleaning and disinfection, and the wear and tear of the instruments.

The implant positional guide is a type of implant guide that improves the precision of implant surgery by controlling the accuracy of the first drill. Unlike surgical guides, the implant positional guide does not require designing different guides for different implant systems and does not require the use of a matching guided surgery cassette, which is universally applicable and compatible. Because it is used only in the first drill, no metal sleeves and no corresponding drill handle are required as well. The implant positional guide can be printed with a model resin material with a certain strength, saving the cost of cleaning and the instrument itself. It is also easy to use. Because with the first precise drill, the operator does not have to check the axial position repeatedly, which simplifies the procedure. Although the implant positional guide only serves to position the implant during the first drilling, implant surgery is very focused on the positional of the first drill. “A good start is half the battle”. The first drill determines the accuracy of the subsequent implant socket preparation. Therefore, all subsequent drilling procedures can be going step by step in the direction of the first drill.

The definition of the accuracy of the implant guide is the positional or angular deviation between the actual and the planned position. Errors can occur from the collection of image data to the final surgical placement of the implant [17, 18]. The matching ratio between the planned and real implant positions was measured by two cone-beam computed tomography (CBCT) or multilayer computed tomography (MSCT) scans, realizing overlap measurements between preoperative planning and postoperative implant position [19]. The deviation between the virtual planning of the implant and the actual in vivo position is always occurs [20].

This study aimed to measure the accuracy of implant placement guided by the implant positional guide and to assess its clinical value in helping clinicians to improve implant surgical precision and save costs in implant surgery.

## Methods

### Participants surveyed

This research was performed in compliance with the World Medical Association Declaration of Helsinki on medical research. The content of this study was approved by the Ethics Committee of West China College of Dentistry (reference number: WCHSIRB-D-2022-457) and informed consent was obtained from the patients.

This study population was patients with a single tooth missing requiring implant restoration who visited the West China Dental Hospital of Sichuan University from April 2023 to May 2023.

The sample size was calculated with the angular deviation refined from a study, which was 4.86 ± 2.10°, and was used as the main outcome variable [21]. With alpha level of 0.05, sample size ratio of 1:1 between the two groups, and a degree of certainty (test efficacy) of 1 − β = 90%. The total sample size required to compare the two implant methods was calculated to be 48 implants (*n* = 24 for each method). All the implants are ITI bone level or bone level tapered type and placed by the same surgeon. Patients chose their implant method based on the specific information such as price, procedure of implant surgery, individual situation and so on given by the doctor about the two implant methods. The group that was implanted using the implant positional guide was the test group, while the control group was implanted by freehand (FH). In order to control the impact from implant placement site and the parameters of the implant, we chose patients who used implants of approximately the same diameter, length and other parameters. In addition, we ensured a 50/50 split between anterior and posterior implants in the test and control groups, i.e., 12 anterior implants and 12 posterior implants out of 24 implants in each group. The information of the implant placement site and the diameter and length of the implant is shown in Tables 1 and 2.

**Table 1:** Implant placement site of the implants

	Freehand				Implant positional guide			
	Anterior implants		Posterior implant		Anterior implants		Posterior implant	
	Teeth site	Number	Teeth site	Number	Parameter	Number	Parameter	Number
Implant placement site	11 12 13 21 22 31 33	3 2 1 1 3 1 1	14 15 16 24 25 26 36 46	1 1 2 2 1 3 1 1	11 12 13 21 22 31	3 1 2 2 3 1	15 16 25 26 36 46	1 3 2 3 1 2

**Table 2:** Length and diameter of the implants

	Freehand				Implant positional guide			
	Anterior implants		Posterior implant		Anterior implants		Posterior implant	
	Teeth site	Number	Teeth site	Number	Parameter	Number	Parameter	Number
Parameter of the implant	ITI3*12 ITI3.3*10 ITI3.3*12	2 3 7	ITI4.1*10 ITI4.1*12 ITI4.8*10	6 1 5	ITI3.3*10 ITI3.3*12 ITI4.1*10 ITI4.1*12	1 5 4 2	ITI4.1*10 ITI4.8*10	4 8

### Inclusion and exclusion criteria

Patients under test must meet the following inclusion and exclusion criteria.

Inclusion criteria: ① healthy adults (18–65 years); ② patients with single missing teeth who require implant restoration without bone augmentation; ③ good oral hygiene: full mouth plaque index ≤ 25%; full mouth bleeding index less than or equal to 25%.

Exclusion criteria: ① patients with an opening of fewer than three fingers, which is not conducive to the placement of a guide; ② patients with missing teeth at the free end.

### Treatment process

**Fig. 1:**
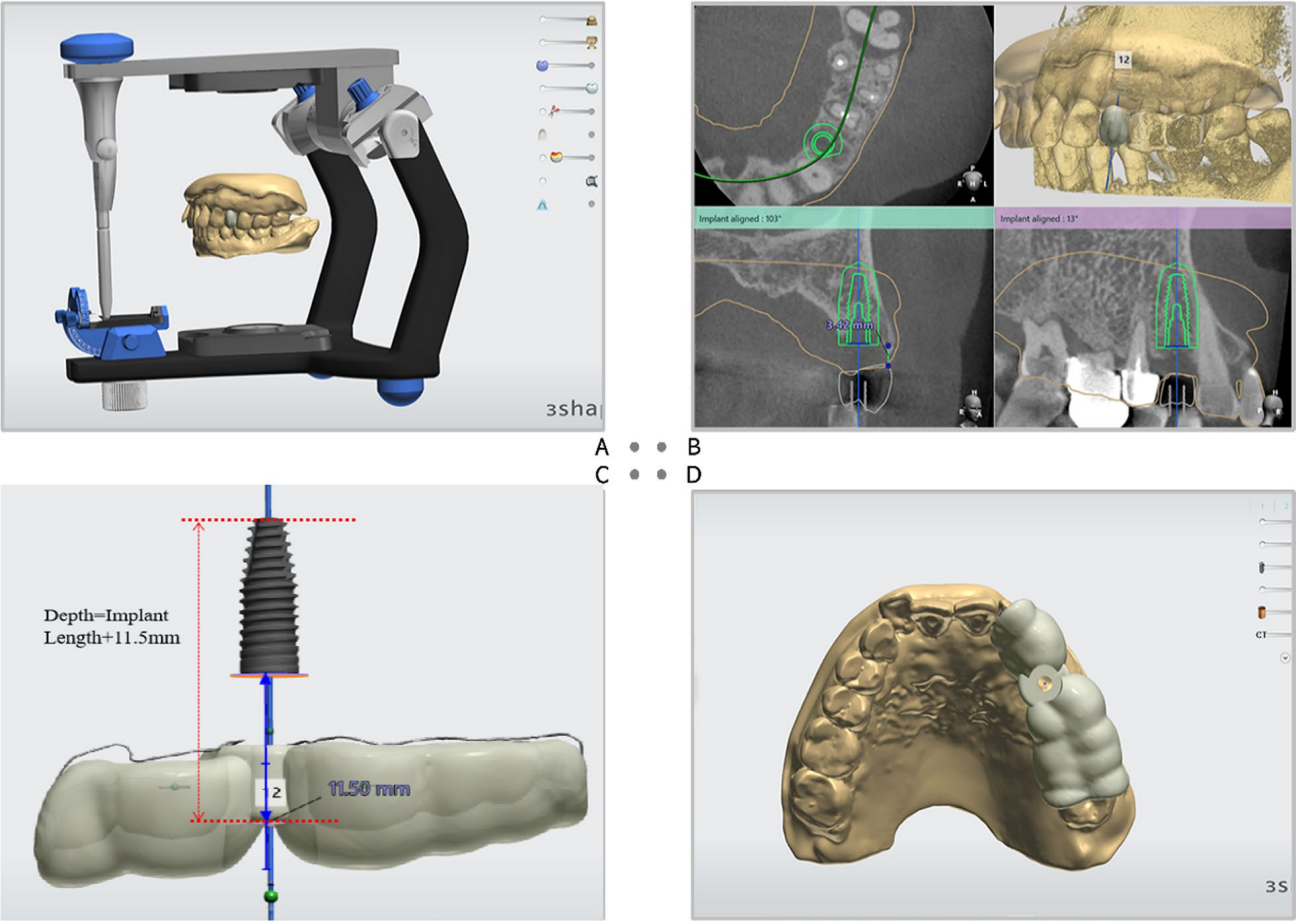
**A** Virtual tooth set-up on the articulator. **B** Implant position planning. **C** Measure the depth from the tip of the implant to the top shoulder of the sleeve. **D** Generate the implant positional guide

**Fig. 2:**
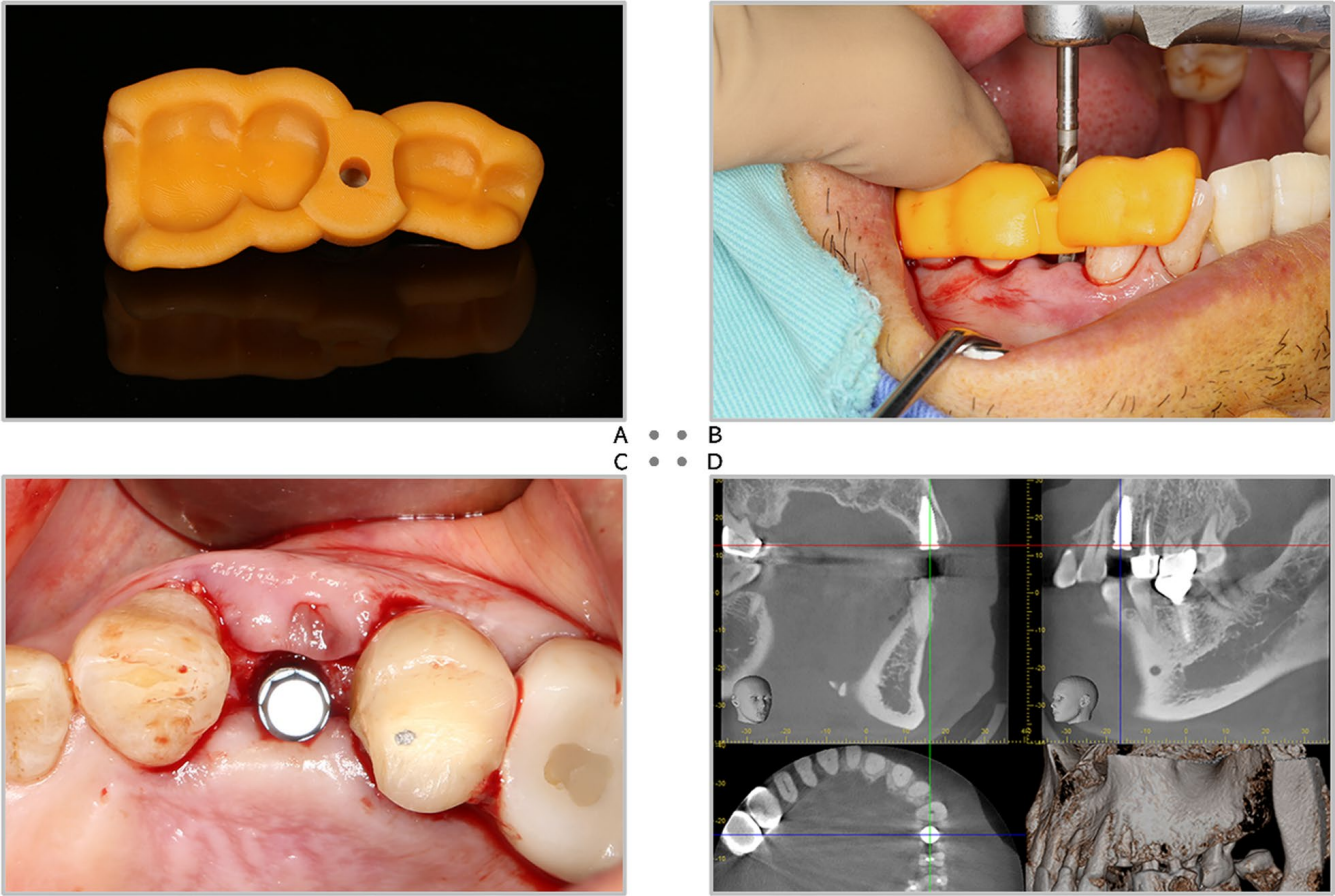
**A** The printed implant positional guide. **B** Firmly seating the guide by fingers and initiate the osteotomy site with a 2.2-mm twist drill. **C** Remove the guide and complete the drilling procedure by freehand. **D** The implant is placed at the planned position

Patients in the trial group were treated according to the following procedures:

1. Load the scanned maxillary and mandibular arches into a commonly used surgical planning software program (Implant Studio; 3Shape A/S), then orient the arches to the virtual articulator and conduct a digital tooth set-up driven by prosthetic considerations (Fig. 1A).
2. Accurately superimpose the scanned arches with the cone-beam computed tomography (CBCT) data, and virtually planning the implant at the most optimal position following the 3A-2B principle [22] and with respect to the anatomical and prosthetic limitations (Fig. 1B).
3. Select a sleeve whose external diameter is 0.2 mm wider than the pilot drill to prevent the drill from contacting the guide during the surgery and ensure an effective deviation restriction at the same time. A third-party software, named Materialise Magics 23.0, is used to adjust the diameter if there are no suitable sleeves. The length of the sleeve is about 6 mm by taking into account both the deviation control and the patient’s mouth opening limitation. The depth from the tip of the implant to the top shoulder of the sleeve is measured (Fig. 1C). Then generate the guide by covering at least two adjacent teeth mesially and distally for stability (Fig. 1D).
4. Print the implant positional guide with a 3-dimensional (3D) printer (UltraCraft A2D 4K) and verify its seating by ensuring there is no gap between the remaining dentition and the occlusal margin of the guide (Fig. 2A, B).
5. Firmly position the guide by fingers or periosteal elevator and use it to initiate the osteotomy site with a 2.2-mm twist drill (Fig. 2B). Then remove the guide and complete the subsequent drilling and implant inserting procedure by freehand (Fig. 2C). Depth control is achieved through the indication lines on the drills according to the preoperative planning.
6. Postoperative CBCT indicates the implant is placed at the planned position (Fig. 2D).

In the control group, the patients were implanted by freehand.

**Fig. 3:**
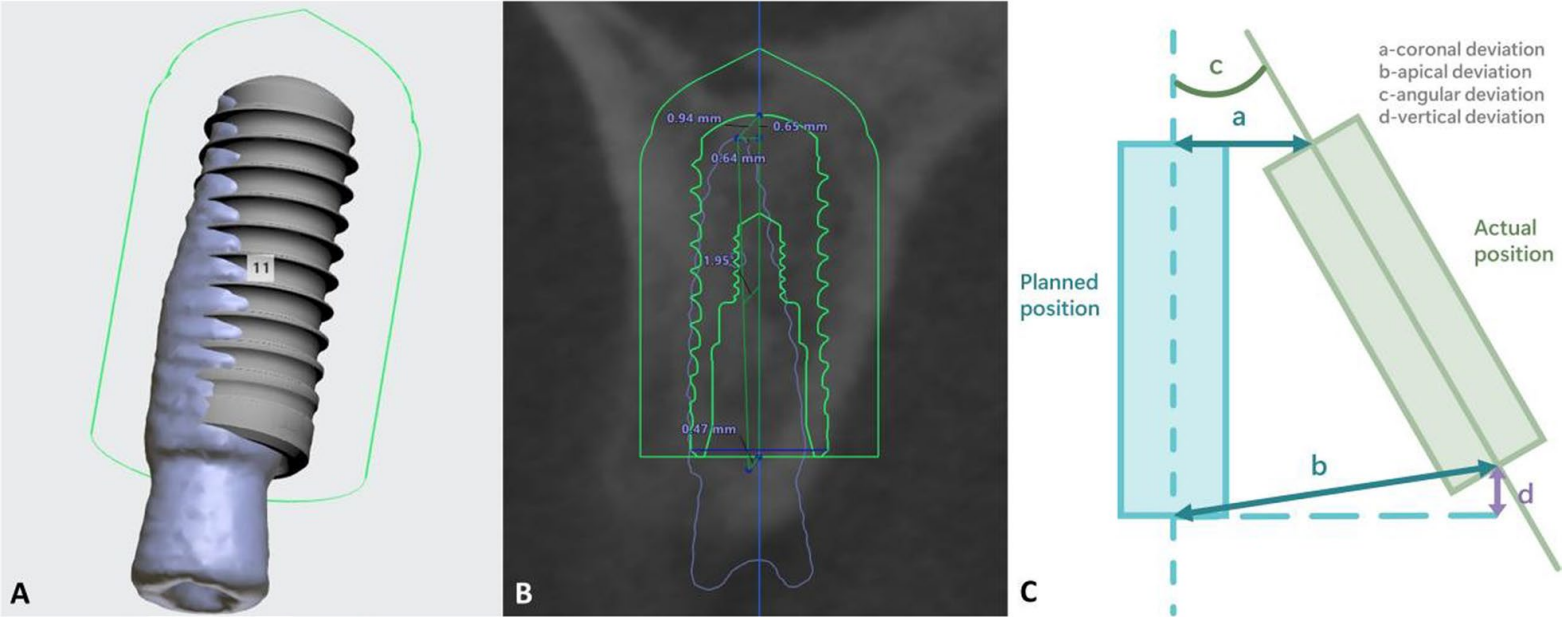
**A** Comparison of the actual and planned 3D position. **B** Measuring the deviation of the actual and planned position. **C** Schematic diagram of deviation measurement of the planned and actual position

**Fig. 4:**
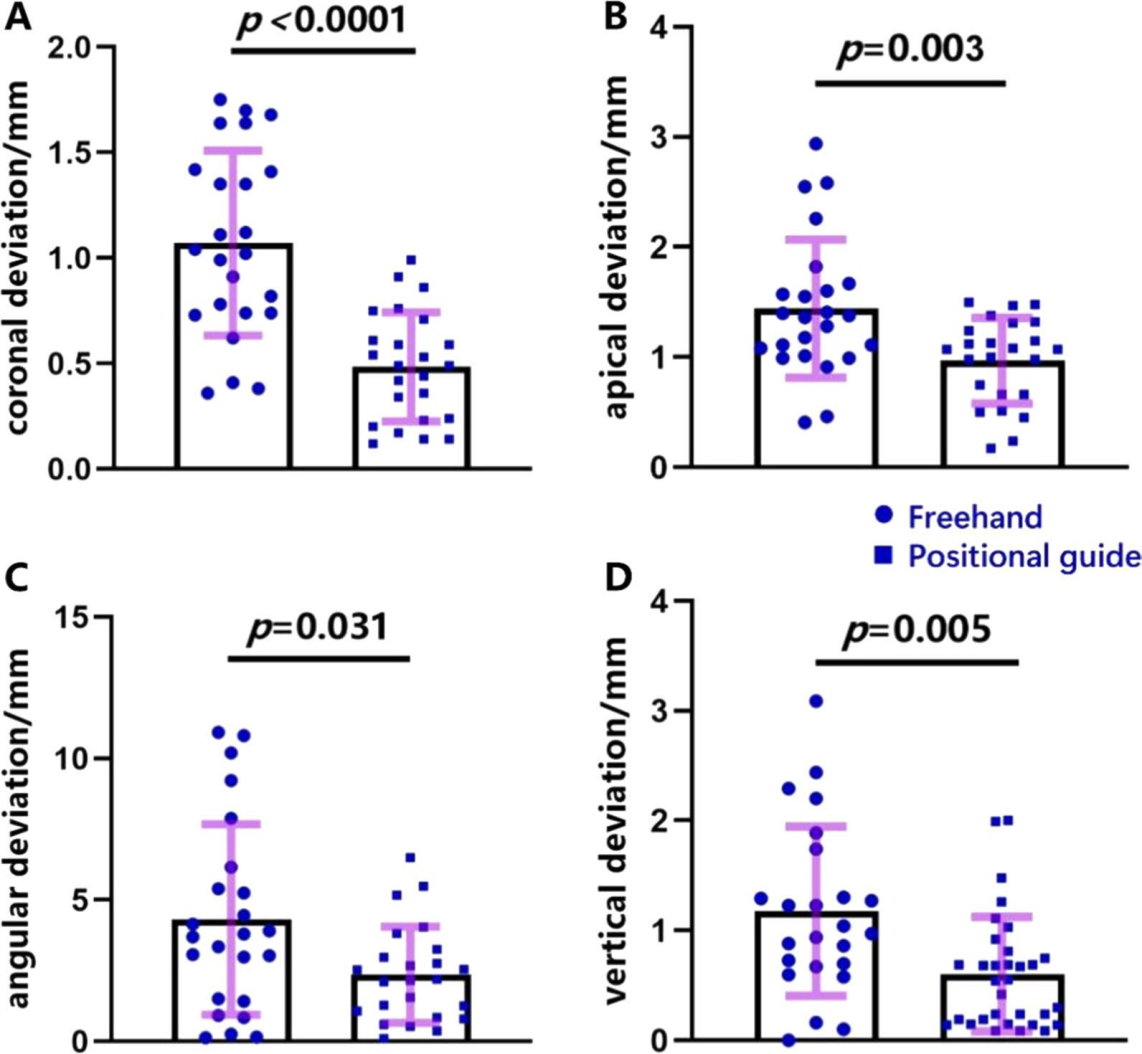
Accuracy comparison

Implant placement accuracy measurements:

1. 3D reconstruction of the postoperative CT using third-party software (Mimics Medical 21.0) to create a 3D model of the tooth and implant and saved in stereo lithography (STL) format.
2. Importing the postoperative 3D model into the surgical planning software program (Implant Studio; 3Shape A/S) (Fig. 3). Align the preoperative and postoperative 3D models with the dental alignment marker points and measure the coronal deviation, apical deviation, angular deviation, and vertical deviation between the actual and the planned position (Fig. 4).

### Statistical analysis

IBM SPSS Statistics for Windows (Version 21.0) was conducted to analyze the data. Descriptive statistics for all the parameters were recorded (coronal deviation, apical deviation, angular deviation, and vertical deviation). Means, standard deviations, and 95%-confidence intervals were calculated for angulation and position deviations. We conducted Mann–Whitney U test to assess the statistical significance of differences between each approach. A *p* value of < 0.05 was considered statistically significant.

## Results

This study included 48 healthy patients of the age group 18–65 years with a single missing tooth who require implant restoration without bone augmentation in the study period. 24 patients were treated with the implant positional guide as the test group and 24 patients were treated by freehand as the control group. All implants were ITI bone level.

Table 3 depicts the deviations of the two groups, including the mean deviation, standard deviation, 95%-confidence interval (Cl), and the *p*-value. In addition, Fig. 4 presents these data in the form of a box-plot diagram. Overall, surgery with an implant positional guide realized a higher accuracy in terms of all parameters describing in the deviation of the actual and planned implant position than freehand implant (Table 3). Compared to the freehand implants, the implant positional guide group reached statistically significant higher accuracy values for coronal deviation (0.48 ± 0.26 vs 1.07 ± 0.44; *p* < 0.0001), apical deviation (0.97 ± 0.39 vs 1.44 ± 0.63; *p* < 0.001), angular deviation (2.36 ± 1.70 vs 4.31 ± 3.37, *p* < 0.05) and vertical deviation (0.60 ± 0.52 vs 1.18 ± 0.77; *p* < 0.01). In addition, we could find that the degree of statistical differences in implant accuracy between the freehand and implant positional guide groups were, in descending order, coronal deviation, apical deviation, vertical deviation, and angular deviation. This suggests that the implant positional guide has a better effect in controlling the coronal position, but is slightly less effective in controlling the implantation angle. Moreover, as shown in Fig. 4, we can find that the accuracy of the freehand group is more dispersed while the accuracy of the implant positioning guide group is more concentrated, which reveals high stability and a small variation of the implant positional guided surgery.

**Table 3:** Deviation of the actual and planned implant position (mean and standard deviation (SD), and 95%-confidence interval (CI)) for implant surgery using an implant positional guide in comparison to freehand implant

Deviation of the actual and planned implant position		Coronal deviation	Apical deviation	Angular deviation	Vertical deviation
Freehand	Mean	1.07	1.44	4.31	1.18
	(SD)	(0.44)	(0.63)	(3.37)	(0.77)
	{95%-CI}	{0.89–1.26}	{1.18–1.71}	{2.89–5.73}	{0.85–1.50}
	Tests of normality	*p* = 0.197 (normal distribution)	*p* = 0.083 (normal distribution)	*p* = 0.029 (does not follow a normal distribution)	*p* = 0.196 (normal distribution)
Implant positional guide	Mean	0.48	0.97	2.36	0.60
	(SD)	(0.26)	(0.39)	(1.70)	(0.52)
	{95%-CI}	{0.38–0.59}	{0.80–1.13}	{1.64–3.08}	{0.42–0.79}
	Tests of normality	*p* = 0.330 (normal distribution)	*p* = 0.135 (normal distribution)	*p* = 0.099 (normal distribution)	*p* = 0.014 (does not follow a normal distribution)
Test of homogeneity of variances		*p* = 0.007 (unequal variances)	*p* = 0.131 (equal variances)	*p* = 0.008 (unequal variances)	*p* = 0.065 (unequal variances)
Mann–Whitney U test		*p* < 0.0001	*p* = 0.0007	*p* = 0.031	*p* = 0.005

## Discussion

This study evaluates the accuracy of implant placement by using an implant positional guide versus freehand. The parameters for assessing deviation included (a) coronal deviation, (b) apical deviation, (c) angular deviation, (d) vertical deviation. In this study, we found an overall degree of deviation was significantly lower in guided surgery with implant positional guide approach than the freehand. From all aspects of compatibility of restoration such as functional, esthetical and biological, implants must be placed correctly in an ideal position. Correct implant position not only has favored prosthetic and esthetic outcomes it has also shown long-term stability of peri-implant hard and soft tissues [23].

By introducing guided surgical protocol, static computer-assisted implant placement (sCAIP) helps clinicians to overcome these difficulties. In sCAIP, a digitally designed surgical guide is used to precisely control osteotomy preparation and implant placement. sCAIP approach shows greater accuracy in implant placement, reduces the necessity of invasive adjunctive procedures and bone augmentation, and prevents patient distress. There are two variations of sCAIP namely fully guided (FG) placement and partially guided(PG) placement [24]. In guided surgery, a surgical guide act as a tool that helps in the transfer of a digital surgical treatment plan to the patient [25]. The implant positional guide simplifies the surgical procedure with the precise position of the first drill, the operator does not have to check the axial repeatedly. It helps the clinician to simplify the surgical procedure starting from the diagnostic phase up to the restoration-oriented design [26], thereby helping in the accurate placement of the implant which results in a commendable esthetic and prosthetic outcome [23].

The observed accuracy of the implant positional guide in comparison to freehand in the present study is similar to other reported studies. According to the study of Jaffar Abduo et al. The FG (fully guided) implant shows less deviation than PG (partially guided) and FH (freehand), while the FH implants appeared to have the greatest deviations and variations in most of the variables. On the other hand, PG placement is more accurate than the FH especially, since the coronal and apical accuracy is at a similar level to the FG placement [24]. A randomized controlled trial implemented by Palita Smitkarn et al. showed the median deviations of surgery guide implants in angles, shoulders, and apexes were 2.8 ± 2.6°, 0.9 ± 0.8 mm, and 1.2 ± 0.9 mm, lower than the implant positional guide group [27]. Although the accuracy of FG is higher than PG and FH due to technological advancement [24], the FG protocol is still prone to error. This error can occur at any step of planning, guide fabrication, and implant placement procedure [25].

Although FG protocol has higher accuracy among PG and FH, it is more costly due to the need for a special kit, and the treatment procedure is time-consuming. The present study shows the use of the implant positional guide is cost-effective because it does not require the design of different surgical guides for different implant systems. It uses only a first drill, a metal sleeves and the corresponding drill handles are not required. M. Tallarico et al. demonstrated that when limited space is available, an implant placed using a surgical guide without metal sleeves is more accurate. The non-metal sleeve of small diameter is recommended to reduce lateral drill movement and instrument tolerance [28]. In a nutshell, by controlling the accuracy of the first drill, the implant positional guide has higher accuracy than freehand implants and has less difficulty handling than a regular surgical guide, which also helps reduce the financial burden on the patients. It is a promising technology that guarantees a low cost and high precision of implant surgery.

While the accuracy of the first drill has a significant impact on the final accuracy of the implants, it cannot be ignored that during surgery, when the implantation cavity is enlarged, the implantation position and direction may gradually shift. We think it is workable to control the final implantation position with the implant positional guide. Due to the low cost of the implant positional guide, we can fabricate another guide to match the final drill to control the final implant position. We expect that this can be confirmed in further clinical trials. In addition, all implant surgeries in this clinical trial were flap surgeries. Flapless surgery using the implant positional guide was not performed. We believe that whether there is a difference between the implant accuracy of flap and flapless implantation procedures can be verified in further clinical trials.

The implant positional guide uses model resin material as the 3D printing material, which not only can guarantee a certain strength but also can greatly reduce the cost. However, the model resin material also has disadvantages. It is less strong and has a shorter storage time compared to common surgical guide instruments. It may also be deformed, affecting the precise position of the implant. In addition, as the implant positional guide does not require a metal sleeve, it may produce contamination during the implantation process. But since the guide only needs to be used at the first drilling, the contamination it produces can be washed away immediately. There are no cases of infection in current clinical practice.

Unlike the precise positional of the implant depth of the surgical guide, the implant positional guide needs to observe the graduated line during the implant procedure to determine the implantation depth. Since the graduated line may be partially obscured by the guide or have a limited field of view in case of insufficient mouth opening, the final vertical deviation values are probably closer to the freehand planting measurements.

Despite the simplicity of the use of the implant positional guide, it has certain limitations such as it cannot be used in complex cases for example in multiple missing teeth and edentulous patients. The use of resin makes the production economical but may compromise the strength so the clinician must be cautious while using it. Due to ethical considerations, the implant method is chosen by the patients, complete randomization cannot be guaranteed and could not ignore the presence of selective bias. In addition, blinding could not be guaranteed due to the large difference between the guide and freehand implant methods, which may have an impact on the comparison of implant accuracy between different implant methods. Therefore, it must be acknowledged that the results of the test are limited.

## Conclusion

The implant positional guide is a technique for performing digital oral implants, which can act as a viable tool for dental implant placement by clinicians. From the present study, it can be concluded that the implant positional guide provides more accurate implant placement than freehand. Compared to surgical guides, it is less difficult to perform implant surgery and it is also more cost-effective than a normal surgical guide, reducing the financial burden on the patients. It is a promising technology that guarantees low cost and high precision in implant surgery.

## Data Availability

The data sets used and/or analyzed during the current study are available from the corresponding author on reasonable request.

## Abbreviations

CBCT: Cone-beam computed tomography
MSCT: Multilayer computed tomography
FG: Fully guided
PG: Partially guided
FH: Free hand
sCAIP: Static computer aided implant placement
3D: Three-dimensional
STL: Stereo lithography
